# Species Diversity of Mosquitoes (Diptera: Culicidae) in Various Coastal Bioecological Regions of Southern Iran

**DOI:** 10.1155/jotm/6289369

**Published:** 2026-05-18

**Authors:** Ebrahim Abbasi, Hamzeh Alipour, Shahyad Azari-Hamidian, Kourosh Azizi, Amirhossein Darabi, Sanaz Amiri, Hasan Bakhshi, Mohammad Darvishi, Mohammad Djaefar Moemenbellah-Fard

**Affiliations:** ^1^ Department of Biology and Control of Disease Vectors, Student Research Committee, School of Health, Shiraz University of Medical Sciences, Shiraz, Iran, sums.ac.ir; ^2^ Department of Biology and Control of Disease Vectors, School of Health, Research Center for Health Sciences, Institute of Health, Shiraz University of Medical Sciences, Shiraz, Iran, sums.ac.ir; ^3^ School of Health, Research Center of Health and Environment, Guilan University of Medical Sciences, Rasht, Iran, gums.ac.ir; ^4^ Department of Medical Parasitology, Mycology and Entomology, School of Medicine, Guilan University of Medical Sciences, Rasht, Iran, gums.ac.ir; ^5^ The Persian Gulf Tropical Medicine Research Center, The Persian Gulf Biomedical Sciences Research Institute, Bushehr University of Medical Sciences, Bushehr, Iran, bpums.ac.ir; ^6^ Department of Epidemiology, Student Research Committee, School of Health, Shiraz University of Medical Sciences, Shiraz, Iran, sums.ac.ir; ^7^ Vector-Borne Diseases Research Center, North Khorasan University of Medical Sciences, Bojnurd, Iran, nkums.ac.ir

**Keywords:** abundance, anophelinae, biodiversity, culicinae, evenness, hill number, species richness

## Abstract

Blood‐feeding mosquitoes (Diptera: Culicidae) impose a major public‐health burden in tropical regions of the world. They transmit many pathogens to humans and domesticated animals. This study investigated the diversity and abundance of mosquito larvae and adults in nine bioecological regions of Bushehr Province, southern Iran, from May to December 2022. Specimens were collected using standard dippers, CDC light traps, pit shelters, and aspirators on human and animal baits alongside key ecological variables such as temperature and habitat type. Diversity was further analyzed using Hill numbers (*q* = 0, 1, 2) through rarefaction and extrapolation with the iNEXT R package, which standardized sampling effort and confirmed that observed richness approached asymptotic values. Nineteen species representing five genera—*Aedes* (41%), *Anopheles* (12%), *Culex* (44.5%), *Culiseta* (1.2%), and *Uranotaenia* (1.2%)—were identified. Diversity analyses yielded mean *α* = 10.1 ± 3.2 species per habitat, β (Whittaker’s index) = 1.8, and *γ* = 19 species for the entire region; Shannon *H*′ = 2.31 ± 0.41, Simpson *D* = 0.44 ± 0.17, and evenness *J* = 0.63 ± 0.18. The dominance index (Cd = *ni*/*N* × 100) indicated *Ae. caspius* s.l. (Cd = 45% of adults, 30% of larvae) as the most preponderant species across habitats. The highest percentage (65.77%) distribution of this species was in the second largest, mainly nonlittoral county of Dashti, with a relatively high (0.451) Simpson Index incorporating both the abundance (biomass) patterns and species richness. The results highlight marked spatial heterogeneity related to temperature, humidity, and habitat type. Routine entomological surveillance and targeted vector‐management strategies are suggested to reduce mosquito‐borne disease risks in southern Iran. Since several collected species are potential vectors of medically important pathogens, regular surveillance, habitat management, and community awareness programs are recommended to mitigate mosquito‐borne disease risks in southern Iran.

## 1. Introduction

Mosquitoes (Diptera: Culicidae) are one of the most insidious hematophagous insect groups, requiring blood from humans and animals to develop their eggs [1]. Various mosquito species thus transmit many pathogens to humans and animals, implicating the importance of region‐specific research to enhance understanding of their ecological roles [2]. Southern Iran encompasses a wide range of coastal and inland bioecological conditions that provide diverse larval habitats and ecological niches for mosquito populations.

Up to now, two culicid subfamilies of Anophelinae and Culicinae, encompassing 41 genera and 3727 species, have been reported worldwide [3]. Currently, 8 genera and 73 species are found in Iran [4, 5]. The most crucial mosquitoes found in Iran belong to the genera *Anopheles*, *Culex*, and *Aedes,* which bite humans and transmit various pathogens. Realizing the multifaceted significance of mosquitoes is paramount to comprehensive ecological management and public health strategies.

Diversity and abundance of mosquitoes rise under optimal ambient conditions of various habitats, which could lead to the reduction of mosquito‐borne diseases. In contrast, a loss in mosquito biodiversity and reservoir hosts may result in a general “dilution effect,” possibly exacerbating vector‐borne zoonotic disease outcomes [6–8]. Three complementary levels of biodiversity were examined. Alpha (α) diversity represents the average species diversity within a single habitat and incorporates both richness and evenness (e.g., Shannon and Simpson indices). Beta (β) diversity quantifies the compositional difference between habitats and was calculated using Whittaker’s index (*β* *w* = *γ*/*α* – 1). Gamma (γ) diversity expresses the total number of species recorded across all habitats in the study area. Evaluating these indices helps identify biodiversity “hotspots” and prioritize habitats for targeted mosquito surveillance and control programs [3, 9]. Studies on mosquito biodiversity indices, such as species richness (the number of species available in a specific local habitat), species evenness (or equitability), and Simpson and Shannon–Wiener indices, are essential to developing vector surveillance and control strategies in different areas [10].

Transmission of malaria and filariasis parasites, multiple arboviruses leading to encephalitis, and biting nuisance have made mosquitoes one of the most deleterious groups of arthropods in public health [1, 10, 11]. At least 15 mosquito‐borne pathogens that cause human and avian malaria (protozoal), avian pox, bovine ephemeral fever, dengue virus (DENV) fever, Rift Valley fever (RVF), Sindbis fever, West Nile virus (WNV), and chikungunya virus (CHIKV) (arboviral) [11], anthrax, tularemia (bacterial), *Deraiophoronema evansi* infection, dirofilariasis, lymphatic filariasis, and setariasis (helminthiases) have been reported in Iran [4, 5].

Based on human seroprevalence surveys, three arboviral fevers, including DENV, CHIKV, and WNV infections, appear to be present in Bushehr Province, though no autochthonous virus transmission by mosquitoes or vector incrimination has been confirmed yet [9]. CHIKV infection was first reported serologically in southeast Iran during 2009–2010 [12], and subsequent evidence from 2022 confirmed human antibodies in several southern provinces, including Hormozgan and Bushehr [9]. Although no autochthonous transmission has yet been documented in Bushehr Province, climatic and trade connections with endemic Persian Gulf regions suggest a potential risk for CHIKV introduction. Moreover, two important veterinary mosquito‐borne pathogens, the bovine ephemeral fever virus and a camel parasitic worm (*Deraiophoronema evansi*), have been reported from this province [4].

Furthermore, five mosquito genera, including *Aedes*, *Anopheles*, *Culex*, *Culiseta*, and *Uranotaenia*, with a total of 30 species, have been found in Bushehr Province [13–22]. Prior knowledge of the relative abundance and frequency of the relevant mosquito species is indispensable to fulfill the needs of an ecologically sound and successful control program. To the best of authors’ knowledge, little information is, however, known about the family of Culicidae in this province despite previous attempts. Lately, 10 different species of *Culex* and four species of *Anopheles* were mainly found in uninhabited areas of the northern parts of this province [23]. Data on mosquitoes and their associated ecologies in this region are scant and need to be substantiated in future research.

The main objective of this study was to assess mosquito species diversity and abundance using α‐, β‐, and γ‐indices across distinct coastal and inland habitats in bioecological regions of Bushehr Province, southern Iran, and to elucidate their ecological and epidemiological significance. In order to provide a foundation for effective vector management and disease surveillance in endemic regions, it is indispensable to understand how ecological heterogeneity, including temperature, humidity, and salinity gradients, affects mosquito community composition. Similar approaches have been employed in recent studies exploring the relationships between mosquito biodiversity, climate variability, and disease transmission in Iran and other arid coastal ecosystems [3, 6].

## 2. Materials and Methods

### 2.1. Study Area

**FIGURE 1:**
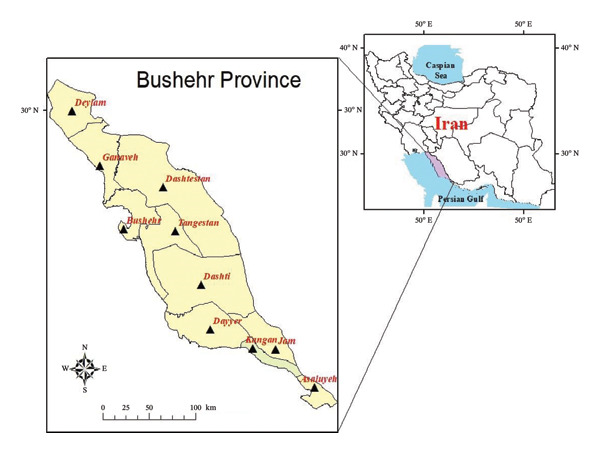
Map of study areas (left box) within Bushehr Province, in the country of Iran (right box). Map generated in ArcGIS 10.8 (ESRI, USA).

Bushehr Province with a 22,743 km^2^ surface area, is located in the southwest of Iran, extending from the coordinates of 27°19′ to 30°16′N and 50°08′ to 52°59′E on the northern littoral zone of the Persian Gulf [24]. This province is bordered by Khuzestan and Kohgiluyeh‐Boyer‐Ahmad from the northwest, Hormozgan from the southeast, Fars from the north, and the Persian Gulf from the south. Bushehr Province (22,743 km^2^) lies in southwestern Iran between 27°19′–30°16′ N and 50°08′–52°59′ E. The mean annual temperature is 25.7°C, the mean annual humidity = 64%, and annual precipitation ≈ 220 mm [25]. Figure 1 represents the location of Bushehr Province, which encompasses Asaluyeh, Kangan, Bushehr, Dashtestan, Dashti, Deylam, Dayyer, Ganaveh, Jam, and Tangestan Counties (Figure 1). The first two counties of Asaluyeh and Kangan were deliberately unified in this research due to their homologous landscape topography harboring petrochemical industrial complexes, relatively small geographic extent, and synchronous prevailing climate conditions.

### 2.2. Sampling Methods and Morphological Identification

Vector sampling was conducted at 18 ecologically representative stations distributed across nine counties of Bushehr Province. Each county contained one fixed sentinel site (sampled continuously throughout the study) and one variable site that was seasonally substituted based on accessibility and habitat type. Thus, a total of 18 stations were established (nine fixed + nine variable). Each larval collection occurred about 15–20 min. These samplings took place from May to December 2022. This cross‐sectional descriptive analysis investigated the mosquito fauna. Some 18 representative stations were chosen based on ecological representativeness (presence of diverse aquatic habitats), accessibility, and logistic feasibility. Each station was sampled during eight rounds (biweekly intervals) to account for seasonal variation.

Larvae were collected from the selected aquatic (freshwater and brackish water) inland habitats by the dipping method using a standard (350 mL capacity) dipper on a biweekly interval basis. All larvae were cleared in lactophenol for 7–10 days and mounted in Canada balsam; adults were pinned and identified using the keys of Azari‐Hamidian and Harbach (2009). Third‐ and fourth‐instar larvae were targeted for identification. Larvae were sampled from a wide variety of aquatic habitats, including irrigation channels, temporary rain pools, animal footprints, rice fields, tree holes, palm irrigation ditches, water storage tanks, and saline pools. Since larval collections focused on natural and seminatural water bodies, artificial containers (e.g., pots, tires, small plastic or metal cans) were excluded. These habitats are known to harbor *Aedes aegypti* and *Ae. albopictus*; therefore, these invasive species may have been undetected in this survey.

Four complementary techniques were used to capture adult mosquitoes: (1) CDC light traps; (2) manual aspirators on human and animal baits; (3) pit shelters; and (4) bed‐net traps. CDC light traps (John W. Hock Co., Model 512) were installed at two fixed locations per station, approximately 1 hour before sunset, and operated overnight (12 h) from dusk to dawn. Trapping was performed twice per month at each site throughout the study period (May–December 2022). Bed‐net and human‐bait collections were restricted to summer and autumn, corresponding to peak adult mosquito activity in southern Iran, to maximize sampling efficiency and reduce ethical and logistical constraints. Aspirator collections (Prokopack, Model 1419, USA) were conducted indoors and outdoors on human volunteers and animal hosts (goat: *Capra hircus* and chicken: *Gallus gallus domesticus*) between 18:00 and 22:00 h—the peak activity period for most local mosquito genera [26].

Each adult sampling session lasted 30 min per method per station. Collections were carried out by trained entomological technicians holding at least a Bachelor of Science degree in medical entomology or public health. To minimize observer bias, all collectors received uniform training, and at least one member of the central team supervised collections in every county. All human landing catches were performed under ethical approval (IR.SUMS.SCHEANUT.REC.1401.121) with informed consent and protective clothing. Pit shelters (1 m depth, shaded) were examined twice monthly, capturing resting mosquitoes during early morning hours. Bed‐net traps baited with humans or animals were set up at one site per county during the summer and autumn months. These traps (untreated white cotton, 1.5 × 1.5 × 1.5 m) were deployed overnight (18:00–06:00 h). Traps were not treated with insecticides; specimens resting inside or on the net were collected by aspirator the following morning.

The adult mosquitoes were also mounted, and the details of their localities and dates of collection were recorded. The third and fourth instar larvae were mounted on microscope slides using Canada balsam as the mounting medium. Microscope slides were examined under a compound light microscope using morphological taxonomic keys [26]. The identification of mosquito species was conducted using the validated keys of Azari‐Hamidian and Harbach (2009), which are widely recognized for the mosquito taxonomy in Iran. The CDC light traps (John W. Hock Co., Model 512) and battery‐operated aspirators (Prokopack, Model 1419, USA) were employed. The use of both adult and larval sampling techniques ensured comprehensive coverage across different habitat types.

### 2.3. Bioecological Regions

Multiple ecologically distinct habitats were encountered during the sampling of larval and adult mosquitoes in Bushehr Province. The second figure comprises these habitats, including arid land, bush land, caves, hedgerows, littoral areas, open fields, palm groves, saline cavities, and woodlands, respectively (Figures 2(a), 2(b), 2(c), 2(d), 2(e),, 2(f), 2(g), 2(h), and 2(i)). The flowing riverine habitat was excluded from this report, as it was dry for most of the year, except during intermittent flush floods. The descriptions of these ecological habitats are as follows:

 Arid land: This habitat is characterized by bioecological adaptations to scarce water sources, temperature extremes, and a unique arid ecosystem, which are important considerations to apprehend the resilience and distribution of mosquitoes in such environments. It explores how mosquitoes thrive in regions with low precipitation (Figure 2(a)).
 Bush land: The bioecological dynamics of mosquitoes are intricately linked to vegetation cover and topographical characteristics. This ecological niche explores how mosquitoes in bushland habitats utilize plant structures for breeding, feeding, and shelter, elucidating their role in the broader ecosystem (Figure 2(b)).
 Cave: Within caves, the bioecology of mosquitoes is narrowly shaped by unique environmental factors such as humidity, temperature, and light conditions. Understanding how mosquitoes adapt to and interact within these subterranean environments is pivotal to grasping their life cycles with potential as pathogen vectors (Figure 2(c)).
 Hedgerow: Within hedgerows, the bioecology of mosquitoes intersects with boundary vegetation. Investigating how hedgerows influence the behavior, breeding patterns, and species diversity of mosquitoes provides insights into the spatial ecology of these vectors (Figure 2(d)).
 Littoral: Exploring the littoral habitat sheds light on how mosquitoes adapt to coastal settings. Factors such as salinity, proximity to water bodies, and vegetation influence the bioecology of mosquitoes in this zone, impacting their prevalence and potential for disease transmission (Figure 2(e)).
 Open field: This includes the bioecological aspects of mosquitoes in expansive, unobstructed areas. Realization of how mosquitoes navigate and exploit open fields contributes to a full understanding of their ecological preferences with potential impact on public health (Figure 2(f)).
 Palm land: The palm land section focuses on the bioecology of mosquitoes associated with palm trees. Examining their breeding preferences and behavioral patterns in this habitat contributes to a nuanced understanding of the ecological niches that facilitate mosquito proliferation (Figure 2(g)).
 Saline cavity: This is a distinct habitat with high salinity that supports few salt‐tolerant species. The mosquito larvae living in such habitats have an osmoregulatory ability to remain isotonic with their surrounding milieu (Figure 2(h)).
 Woodland: Woodland habitats contribute significantly to mosquito bioecology. This section explores how mosquitoes interact with the diverse flora/fauna within woodlands, emphasizing their ecological roles and potential implications for disease transmission dynamics (Figure 2(i)).

FIGURE 2Ecological habitats encountered in the present investigation for sampling mosquitoes in Bushehr Province, 2022. (a) Arid land, (b) bush land, (c) cave, (d) hedgerow, (e) littoral, (f) open field, (g) palm land, (h) saline cavity, and (i) woodland (original photographs).

**Figure:**
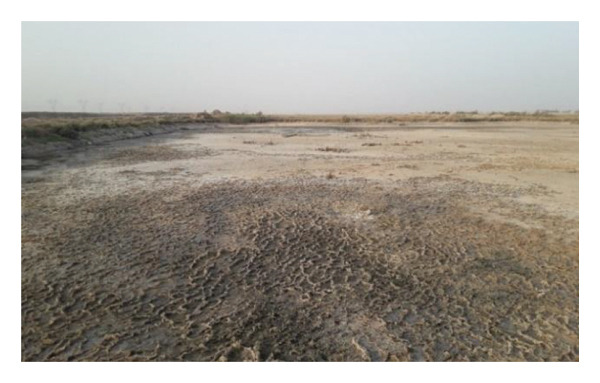
(a)

**Figure:**
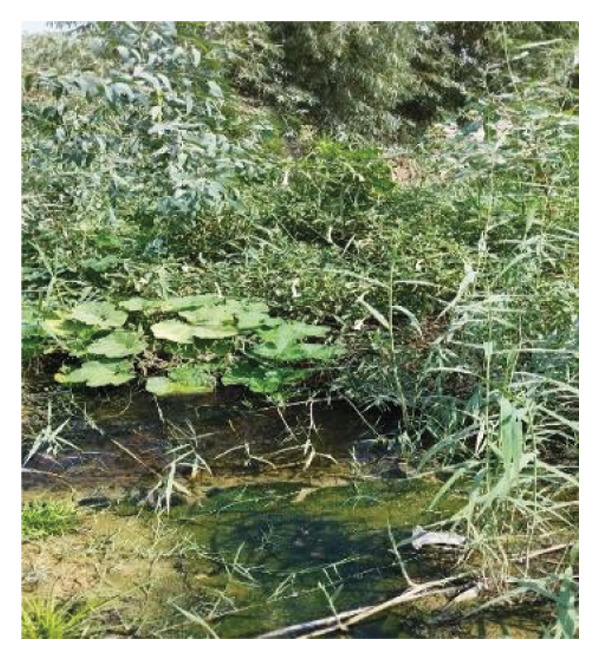
(b)

**Figure:**
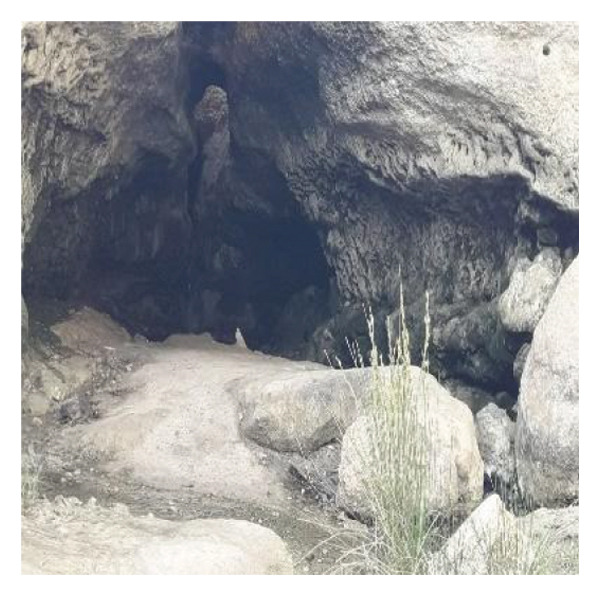
(c)

**Figure:**
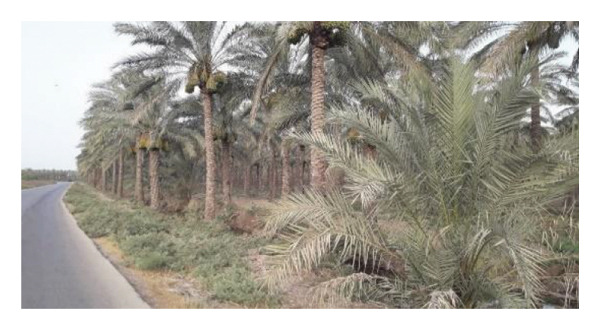
(d)

**Figure:**
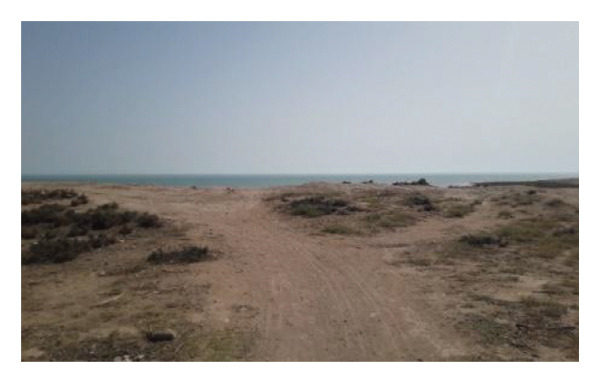
(e)

**Figure:**
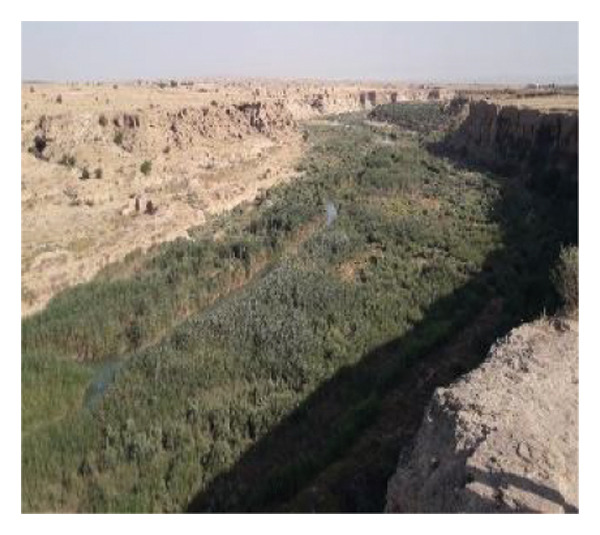
(f)

**Figure:**
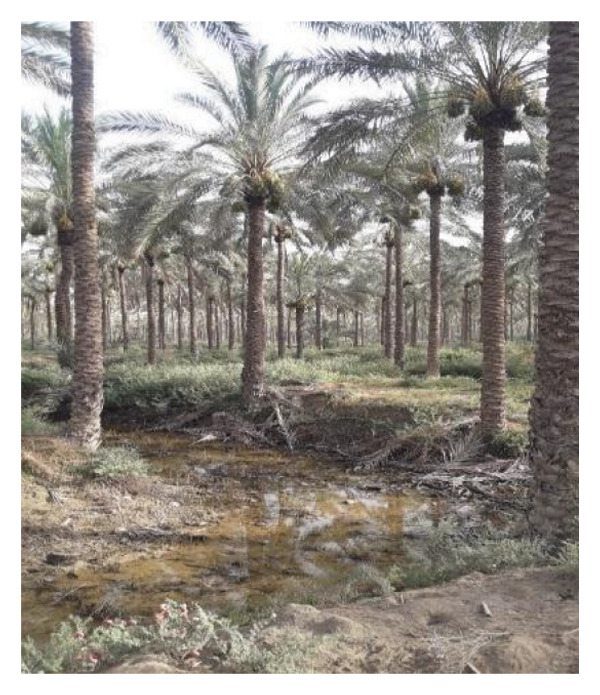
(g)

**Figure:**
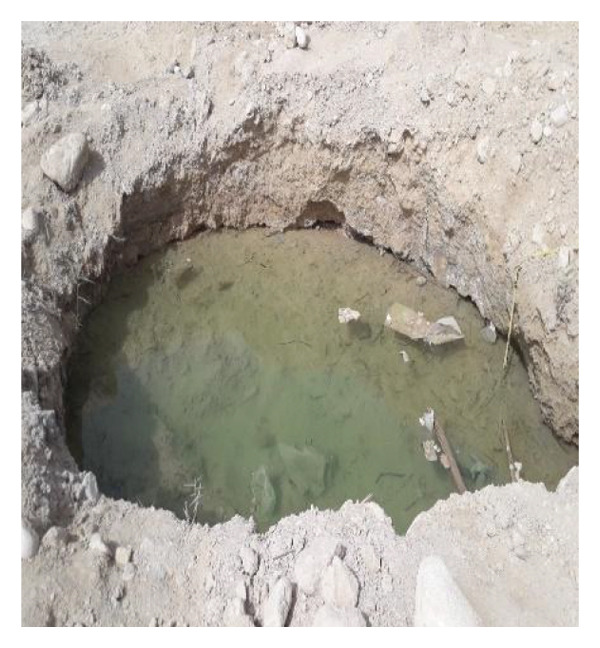
(h)

**Figure:**
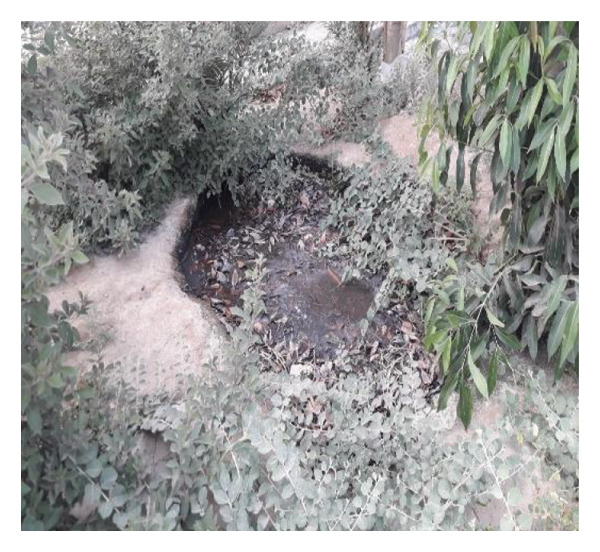
(i)

### 2.4. Biodiversity Indices

Alpha diversity was quantified using Shannon (*H*′) and Simpson (*D*) indices; β diversity was estimated with Whittaker’s index (*β* *w* = *γ*/*α* – 1); and γ diversity equaled the cumulative species richness across all habitats. Evenness (*J*) = *H*′/ln *S*. All indices were computed in PAST v.4.14. Beta diversity is the richness of species encompassing the sum of all the different allopatric species between two specific dissimilar habitats, such as a pond and an open field, whereas gamma diversity is the richness of species across a whole range of habitats within a geographical area in widely separated localities. It is the sum of all the different species present in all the surveyed habitats within a region. Evenness *E* = *H*/(ln*S*) (where *S* is the species richness and *H* is the Shannon index calculated as below) describes the relative abundance of the different species in a region. It ranges between 0 and 1. The Simpson index (*D*) represented by $1÷\Sigma p_{i}^{2}$, where *p*_*i*_ is the proportion of individuals for each species that it contributes to the total in the sample, that is: *p*_*i*_ = *n*_*i*_/*N* (*n*_*i*_ is the number of individuals of taxon *i*, *N* = total number of individuals). This index at zero shows infinite diversity and at 1 reflects no diversity [27]. The Shannon–Wiener index $H=-\sum_{i=1}^{s}Pi*\ln Pi$ indicates species biodiversity in an ecological area. Gamma diversity was applied at the provincial scale to characterize the total regional species pool across ecologically heterogeneous habitats, an approach widely used in regional‐scale biodiversity assessments.

### 2.5. Hill Number–Based Diversity Analyses

To standardize sampling effort and account for rare species, we applied Hill number–based diversity profiles using the iNEXT and vegan packages (R Version 4.3.2). Hill numbers quantify effective species diversity at different orders *q*, corresponding to: – *q* = 0 (species richness, sensitive to rare species), –*q* = 1 (Shannon diversity, equal to exp *H*′), and –*q* = 2 (Simpson diversity, emphasizing dominant species).

Rarefaction and extrapolation curves were computed with 100 bootstraps, following previous reports [28–30]. The analyses allowed direct comparison among sampling stations by standardizing to equal sample completeness (coverage = 0.95). These indices complement traditional α, β, and γ diversity calculations and reduce collector‐effort bias.

### 2.6. Statistical Analysis

To compare outcomes in different groups, one‐way ANOVA and Poisson regression analyses were used. The probability value *P* was taken as 0.05. The data were finally analyzed using SPSS software Version 27. One‐way ANOVA was used to compare diversity indices among counties, while Poisson regression tested differences in species counts. These tests were chosen due to the count‐based nature of entomological data and the need to control for overdispersion and variance differences across sampling sites.

## 3. Results

The results are summarized in both tabular and pictorial forms. A total number of 13,329 specimens of mosquitoes were collected, including 3496 third and fourth‐instar larvae and 9833 female adults. Of all specimens, 46% were collected by CDC light traps, 28% by aspirators (human/animal bait), 18% by standard dippers, and 8% by pit shelters. The species composition varied by method: *Ae. caspius* s.l. Pallas 1771 dominated light‐trap collections, whereas *Cx. pipiens* Linnaeus 1758 and *Cx. quinquefasciatus* Say 1823 predominated in pit shelters and aspirator catches.

The highest number of samples (larvae and female adults) was captured in the largest county of Dashtestan (21%), and the lowest number was present in the littoral Ganaveh (5.67%) (Table 1). The most (14.4%) and the least (4.88%) abundant samples were collected in May and July, respectively. The number of species in each county, separated by larvae and adults, is shown in Table 1. The county of Jam represented the highest values for both evenness (0.840) and the Shannon index (2.474). The county of Tangestan exhibited the lowest (0.0189) the Simpson index (Table 1). In relation to mosquito bioecology, the county of Bushehr, being the capital with the most populated part of the province, had the most biodiverse localities, comprising 7 distinct bioecotypes, while the three counties of Deylam, Dayyer, and Jam were the least biodiverse regions (Table 1). Detailed statistical results included *F* (8,162) = 5.84, *p* < 0.001 for Shannon diversity among counties, indicating significant variation in mosquito biodiversity. Effect size (*η* ^2^ = 0.22) suggested a moderate ecological influence on species distribution. Using one‐way ANOVAR and Poisson regression analyses to compare the data in different groups, significant differences were found between them (*p* < 0.001).

**TABLE 1:** The number of larvae and female adults of mosquitoes collected from each county of Bushehr Province, 2022.

Counties	Topography	Altitude (m)	Larvae (%)	Adults (%)	Total no. (%)	Geographic coordinates	Shannon	Evenness	Simpson	Species richness	1‐D	Bioecology^∗^
Asaluyeh/Kangan	Coastal Plain	Asal. 18 Kan. 5	284 (8.12)	970 (9.87)	1254 (9.41)	27°28′28″N 52°36′41″E 27°50′20″N 52°03′43″E	2.381	0.808	0.1398	11	0.8602	L, A, H, B, O

Bushehr	Coastal Plain Foothill	18	325 (9.30)	1211 (12.32)	1536 (11.52)	28°55′35″N 50°51′05″E	2.166	0.735	0.387	13	0.613	L, P, C, H, W, B, S

Dashtestan	Foothill Plain	80	772 (22.08)	2031 (20.65)	2803 (21.03)	29°17′55″N 51°14′45″E	1.396	0.474	0.449	15	0.550	P, C, H, W, B

Dashti	Coastal Plain	10	497 (14.22)	1501 (15.27)	1998 (14.99)	28°25′N 51°37′E	1.386	0.470	0.451	12	0.549	L, P, C, H, W, B

Deylam	Coastal Plain	5	329 (9.41)	968 (9.84)	1297 (9.73)	30°00′N 50°22′E	2.407	0.817	0.132	10	0.868	L, A, H

Dayyer	Coastal Plain	3	427 (12.21)	713 (7.25)	1140 (8.55)	28°01′N 51°42′E	2.445	0.830	0.121	13	0.878	L, A, O

Ganaveh	Coastal Plain	5	143 (4.09)	613 (6.23)	756 (5.67)	29°29′N 50°38′E	2.360	0.801	0.135	9	0.864	L, A, P, W, B

Jam	Mountainous Plain	675	355 (10.15)	609 (6.19)	964 (7.23)	27°49′25″N 52°19′49″E	2.474	0.840	0.119	14	0.880	P, H, O

Tangestan	Coastal Plain Mountainous	65	364 (10.41)	1217 (12.38)	1581 (11.86)	28°54′14″N 51°21′52″E	1.384	0.470	0.0189	12	0.981	L, C, H, W, O, S

Total			3496 (100)	9833 (100)	13329 (100)	—						—

*p* value			< 0.001^1^	< 0.001^1^	< 0.001^1^		0.003^2^	< 0.001^2^	< 0.001^2^		0.003^2^	

^∗^C, cave; B, bushland; P, palm land; L, littoral; A, arid; H, hedgerow; W, woodland; O, open field; S, saline cavity habitats.

^1^ *p* value for Poisson regression.

^2^ *p* value for ANOVA test.

The rarefaction and extrapolation data indicated near‐complete sampling across counties, with coverage ≥ 0.94. Effective species numbers derived from Hill numbers were *q* = 0 = 19 species (richness), *q* = 1 = 9.8 species (Shannon diversity), and *q* = 2 = 6.2 species (Simpson diversity). These results confirm high evenness in Jam and Deylam and dominance by *Ae. caspius* s.l. in Tangestan. Rare‐species contributions (e.g., *Ur. unguiculata*, *Cs. subochrea*) remained < 2% of total abundance but influenced *q* = 0 estimates.

Species richness values reported in Table 1 represent local (α) richness, defined as the total number of mosquito species recorded within each individual county, pooling larval and adult collections. In contrast, Table 2 presents the cumulative species composition and abundance across all counties, corresponding to regional (γ) diversity for the entire province.

**TABLE 2:** The frequency and percentage distribution of different larvae and female adults of mosquito species collected from each county of Bushehr Province, 2022.

Spp./county		Asaluyeh/Kangan	Bushehr	Dashtestan	Dashti	Deylam	Dayyer	Ganaveh	Jam	Tangestan	Total	%	*p* value
*Ae. caspius* s.l.	Adults	88	845	1408	1108	79	69	43	68	709	4417	44.92	< 0.001
	Larvae	26	83	433	206	27	35	17	31	194	1052	30.09	< 0.001

*An. apoci*	Adults	16	13	21	4	57	6	9	23	39	188	1.91	< 0.001
	Larvae	12	6	16	11	14	7	2	15	18	101	2.89	0.035

*An. dthali*	Adults	20	6	19	17	49	14	7	31	16	179	1.82	< 0.001
	Larvae	14	18	7	14	11	8	2	21	7	102	2.92	0.006

*An. stephensi*	Adults	26	3	25	16	15	36	14	28	26	189	1.92	< 0.001
	Larvae	16	15	21	15	3	22	4	19	15	110	3.15	0.008

*An. subpictus* s.l.	Adults	15	10	35	22	41	34	5	9	27	198	2.01	< 0.001
	Larvae	7	10	12	8	13	23	5	25	13	116	3.32	0.001

*An. superpictus* s.l.	Adults	32	15	23	35	51	48	8	34	39	285	2.90	< 0.001
	Larvae	19	8	21	23	14	22	3	23	11	144	4.12	0.004

*Cx. hortensis*	Adults	13	3	14	8	22	10	17	12	7	106	1.08	0.001
	Larvae	8	15	11	4	6	12	4	21	3	84	2.40	0.001

*Cx. laticinctus*	Adults	23	5	10	5	8	10	19	24	8	112	1.14	< 0.001
	Larvae	14	8	4	5	2	7	4	11	3	58	1.66	0.029

*Cx. mimeticus*	Adults	10	8	6	11	34	9	14	14	8	114	1.16	< 0.001
	Larvae	6	11	8	7	7	9	6	10	2	66	1.89	0.55

*Cx. modestus*	Adults	25	6	16	8	13	8	28	13	2	119	1.21	< 0.001
	Larvae	18	7	6	8	2	6	7	14	1	69	1.97	0.003

*Cx. perexiguus*	Adults	16	21	13	6	24	12	8	12	14	126	1.28	0.026
	Larvae	10	17	7	3	5	11	3	23	5	84	2.40	< 0.001

*Cx. pipiens*	Adults	312	133	198	107	238	206	192	142	159	1687	17.16	< 0.001
	Larvae	34	44	84	92	125	90	19	49	38	575	16.45	< 0.001

*Cx. pusilus*	Adults	44	12	29	14	30	44	43	17	15	248	2.52	< 0.001
	Larvae	28	14	24	26	9	36	21	13	6	177	5.06	< 0.001

*Cx. quinquefasciatus*	Adults	234	95	150	111	179	129	115	124	117	1254	12.75	< 0.001
	Larvae	27	42	74	48	54	53	20	25	32	375	10.73	< 0.001

*Cx. sinaiticus*	Adults	22	10	11	6	38	31	36	12	8	174	1.77	< 0.001
	Larvae	6	8	6	5	6	28	11	23	3	96	2.75	< 0.001

*Cx. theileri*	Adults	22	4	9	8	10	8	26	8	6	101	1.03	< 0.001
	Larvae	13	5	4	9	3	14	6	9	2	65	1.86	0.02

*Cx. tritaeniorhynchus*	Adults	23	4	17	10	29	8	22	18	10	141	1.43	< 0.001
	Larvae	12	3	14	8	13	11	4	15	7	87	2.49	0.81

*Cs. subochrea*	Adults	14	9	20	1	21	22	4	7	4	102	1.04	< 0.001
	Larvae	3	3	11	2	7	21	3	3	2	55	1.57	< 0.001

*Ur. unguiculata*	Adults	15	9	7	4	30	9	3	8	3	88	0.89	< 0.001
	Larvae	11	8	9	3	8	12	2	5	2	60	1.72	0.06

Specific Sum	Adults	970	1211	2031	1501	968	713	613	609	1217	9833	73.77	—
	Larvae	284	325	772	497	329	427	143	355	364	3496	26.23	—

Total (adlt + larv)	—	1254	1536	2803	1998	1297	1140	756	964	1581	13329	100	—

*p* value (Poisson)	—	< 0.001	< 0.001	< 0.001	< 0.001	< 0.001	< 0.001	< 0.001	< 0.001	< 0.001	< 0.001	—	—

%	—	9.41	11.52	21.03	15.00	9.73	8.55	5.67	7.23	11.86	100	—	—

A total of 19 species across five genera of mosquitoes were identified, namely, *Aedes caspius* s.l., *Anopheles apoci, An. dthali, An. stephensi, An. subpictus* s.l.*, An. superpictus* s.l., *Culex hortensis, Cx. laticinctus, Cx. mimeticus*, *Cx. modestus*, *Cx. perexiguus, Cx. pipiens*, *Cx. pusillus, Cx. quinquefasciatus, Cx. sinaiticus, Cx. theileri*, *Cx. tritaeniorhynchus*, *Culiseta subochrea*, and *Uranotaenia unguiculata*. Following species identification (Table 2), the genera of *Aedes* (41%), *Anopheles* (12%), *Culex* (44.5%), *Culiseta* (1.2%) and *Uranotaenia* (1.2%), were subsequently approved by an independent expert on mosquito taxonomy.

Among all species, *Ae. caspius* s.l. was the most frequent (45% female adults, 30% larvae) recorded in every habitat of all counties in Bushehr Province, while *Ur. unguiculata* was the least frequent (1.2%) (Table 2). Most of the *Ae. caspius* s.l. (33.66%) among all counties were found in the nonlittoral Dashtestan, the widest county in this province, while the littoral Ganaveh had the least abundance (1%) of this species. The second most frequent (17%) species was *Cx. pipiens*. This species was very frequent (16%) in Deylam, but the least abundant (7.8%) in Bushehr County. In the counties of Dashti and Tangestan, only three individuals of *Cs. subochrea* and *Cx. modestus* were captured, respectively, representing the lowest number among all caught species in all counties of Bushehr Province. The evenness indices for Dashti and Tangestan (both *J* = 0.47) were equal, indicating similarly skewed species distributions. The number of mosquito species caught in different habitats across all counties in Bushehr Province is shown in Table 2. There were significant differences between the disparate groups (mostly *p* < 0.001), except for the larvae of *Cx. mimeticus, Cx. tritaeniorhynchus*, and *Ur. unguiculata*.

The biodiversity indices indicated that woodland and littoral zones were the most speciose areas (alpha diversity: 18 and 16, respectively), while the saline cavity habitat was the least speciose (alpha diversity: 2) (Table 3). Likewise, the beta diversity between woodland and saline habitats was the largest value among all areas, while the smallest beta diversity value belonged to that of littoral versus woodland areas (Table 3).

**TABLE 3:** Alpha, beta, and gamma diversity indices of mosquitoes in Bushehr Province, Iran, 2022.

Species diversity	Cave	Bushland	Palm land	Littoral	Arid	Hedgerow	Woodland	Open field	Saline cavity
*Ae. caspius* s.l.		√	√			√	√		√
*An. apoci*					√			√	
*An. dthali*	√	√	√	√	√	√	√	√	
*An. stephensi*	√	√	√	√	√	√	√	√	√
*An. subpictus* s.l.		√	√		√	√	√		
*An. superpictus* s.l.	√	√	√	√		√	√		
*Cx. hortensis*	√		√	√		√	√		
*Cx. laticinctus*	√		√	√			√		
*Cx. mimeticus*	√		√	√			√		
*Cx. modestus*	√		√	√			√		
*Cx. perexiguus*	√			√		√	√		
*Cx. pipiens*	√	√	√	√	√	√	√	√	
*Cx. pusilus*	√	√	√	√		√	√	√	
*Cx. quinquefasciatus*	√			√	√		√		
*Cx. sinaiticus*	√			√			√		
*Cx. theileri*	√			√	√		√		
*Cx. tritaeniorhynchus*				√	√		√		
*Cu. subochrea*				√			√	√	
*Ur. unguiculata*				√			√	√	

Alpha diversity	13	7	11	16	8	9	18	7	2
Beta diversity	^∗^C vs. B: 10 C vs. P: 6 C vs. L: 3 C vs. A: 11 C vs. H: 8 C vs. W: 5 C vs. O: 11 C vs. S: 13	B vs. P: 4 B vs. L: 13 B vs. A: 7 B vs. H: 3 B vs. W: 11 B vs. O: 7 B vs. S: 7	P vs. L: 9 P vs. A: 11 P vs. H: 4 P vs. W: 7 P vs. O: 10 P vs. S: 11	L vs. A: 12 L vs. H: 11 L vs. W: 2 L vs. O: 10 L vs. S: 16	A vs. H: 9 A vs. W: 12 A vs. O: 7 A vs. S: 6	H vs. W: 9 H vs. O: 8 H vs. S: 9	W vs. O: 13 W vs. S: 18	O vs*.* S: 5	—
Gamma diversity	19								

*Note:* β diversity = Whittaker’s index (βw), γ diversity = total species richness represents γ diversity (cumulative richness across all counties).

^∗^C, cave; B, bushland; P, palm land; L, littoral; A., arid; H, hedgerow; W, woodland; O, open field; S, saline cavity habitat.

The highest percentage (65.77%) distribution for *Ae. caspius* s.l. among all mosquito species was unveiled in Dashti, the second largest county, while the lowest value (7.94%) was found in Ganaveh. Among anopheline species, the highest distribution value (6.14%) for *An. superpictus* s.l. was in the littoral county of Dayyer, whereas the lowest percentage (1.2%) was recorded for *An. dthali* in Ganaveh. For *Culex* species, *Cx. pipiens* had the largest distribution value (28%) in the northwest littoral county of Deylam.

## 4. Discussion

The present study investigated determining alpha, beta, gamma, Simpson, evenness, and Shannon diversity indices and species distribution in different counties of Bushehr Province. In total, 3496 larvae and 9833 adult females of mosquitoes belonging to 19 species across five genera were collected, among which there were several medico‐veterinary important vectors of arboviruses, some of which have previously been recorded in this area [23]. This data represented 26% of the total mosquito species (73 species) and 62.5% of the total mosquito genera (8 genera) recorded in Iran. The diversity indices derived here, Shannon H′, Simpson D, and evenness J, represent α diversity measures, while Whittaker’s βw captures inter‐habitat turnover. These metrics jointly describe ecological heterogeneity across the province.

Incorporating Hill number diversity profiles (*q* = 0, 1, 2) provided a robust framework to compare mosquito communities under standardized sampling effort. These analyses, which extend beyond the traditional Shannon–Wiener approach, revealed that while overall species richness (*q* = 0) was high across the province, effective diversity (*q* = 1, 2) decreased sharply in saline and arid habitats dominated by a few abundant species. This pattern underscores the influence of environmental filtering on community structure.

Among adult‐collection methods, CDC light traps were most efficient for capturing *Culex* species, particularly *Cx. pipiens* and *Cx. quinquefasciatus*, while aspirator collections on animal baits yielded the highest numbers of *Aedes caspius* s.l. and *Ae. vexans*. Pit shelters were effective for *Anopheles stephensi* and *An. dthali* resting adults. Regarding larval habitats, *Ae. caspius* s.l. and *Cx. pipiens* predominated in brackish and semisaline waters, whereas *Anopheles* larvae were found mainly in clear freshwater habitats such as irrigation channels and palm groves.

### 4.1. Comparison With Neighboring Provinces

In a previous study in the northern parts of this province, only four different species of *Anopheles* and 10 *Culex* species were identified [23]. *Anopheles apoci* was the only anopheline species unreported in the previous study. While our study revealed the presence of *Cx. pusillus* and *Cx. quinquefasciatus*, which were absent from the previous report, the doubtful existence of *Cx. torrentium* needs to be verified due to the probable misidentification of *Cx. pipiens* or *Cx. quinquefasciatus* as *Cx. torrentium*. The α diversity variation among counties (H′ 1.38–2.47) indicated that heterogeneous habitats with mixed vegetation and microclimatic variation supported higher mosquito richness, whereas more uniform saline or arid habitats harbored fewer species.

In a study from the west of adjacent Fars Province to the north of Bushehr Province, the fauna and seasonal activity of mosquitoes were examined, which showed the presence of six species across three genera [31]. The most abundant species in their study was *Cx. theileri*, which contrasts with our findings that identified *Cx. pipiens* as the most abundant *Culex* species (17%). Another study conducted in six southeastern counties of the same province identified 17 different mosquito species across four genera, including 10 *Culex* species and four *Anopheles* species [32]. The preponderant mosquito species was *Cx. pipiens* (27.3%) in agreement with the present study related to *Culex* species. They found the highest species diversity in Mohr County of Fars Province [32]. Considering that this county is almost adjacent to Jam County in the current research, it will be logical to find that their Shannon Index (Mohr, 1.7; Jam, 2.474) approaches each other, reflecting high species richness.

In a study to identify the fauna of mosquitoes of the coastal Chabahar County in Sistan and Baluchistan Province, southeast Iran, next to Pakistan, some 17 species across four genera, including 12 *Culex* species, were outlined [33]. Their pyrethroid space spray collections (PSSC) showed that *Cs. longiareolata* was preponderant (22.8% adults) among all caught species [33].

In the coastal Hormozgan Province, to the east of Bushehr Province, researchers sampling from only four counties identified three genera encompassing 16 species of mosquitoes [34]. The number of different *Culex* species (11) reported in this study was identical to that found in the current survey. In another recent study from the same province, mosquito collections from eight out of 13 counties likewise yielded 16 female culicine species across three genera, 12 of which (75%) were *Culex* species [35]. They identified the three species of *Cx. antennatus, Cx. sitiens*, and *Cx. perexiguus* (as *Cx. univittatus*) as absent from the current study. In reverse, the two species of *Cx. hortensis* and *Cx. laticinctus* in the present research were unreported from their study. Apart from *Ae. caspius* s.l., they also collected *Ae. vexans*, absent from this investigation but reported from other nearby regions. A recent study [36] demonstrated that the *Ae. vexans* of an earlier report [37] was actually that of *Ae. caballus*. While both these surveys found *Cs. subochrea* in synchrony, their collection of *Cs. longiareolata* deviated again from the present survey [35].

### 4.2. Ecological Interpretation of Diversity Indices

This report on mosquito diversity across all counties in Bushehr Province has not been previously investigated; thus, it aimed to outline alpha and beta diversity indices, species richness, and species distribution in different parts of the province. In line with the findings from Sirjan County, Kerman Province, SE Iran, the present mosquito fauna represented 26% of the total number of mosquito species (73 species) recorded in Iran. A different species from the genus *Aedes*, *Ae. vittatus,* accounted for about 2% of those captured in Sirjan County [2]. The diversity indices derived here, Shannon H′, Simpson D, and evenness J, represent α diversity measures, while Whittaker’s βw captures inter‐habitat turnover. These metrics jointly describe ecological heterogeneity across the province. The α diversity variation among counties (H′ 1.38–2.47) indicates that heterogeneous habitats with mixed vegetation and microclimatic variation support higher mosquito richness, whereas more uniform saline or arid habitats harbor fewer species.

### 4.3. Vector and Invasive Species Significance

As stated above, there are currently five genera and 30 species of mosquitoes in the coastal Bushehr Province. Our research found only 19 species (63%) in this province. According to the available reported data, 11 out of the 30 previously identified species, including *Ae. aegypti*, *An. culicifacies* s.l., *An. fluviatilis* s.l., *An. multicolor*, *An. pulcherrimus*, *An. sergentii*, *An. turkhudi*, *Cx. arbeeni*, *Cx. bitaeniorhynchus*, *Cx. deserticola*, and *Cs. longiareolata*, which were recorded in Bushehr Province before, were not discovered during this study. The important invasive mosquito *Ae. aegypti* was recorded for the first time in Bushehr Province by Dow in 1953 [15]. Recently, this species was found in this and its eastern province (Dr. Raeisi, pers. comm., [38]). Lotfi [13] reported *Cx. impudicus* and *Cx. vagans* in Bushehr Province, but his identification was based on unreliable characters, and there are no available verified specimens of them in Iran. Hence, their occurrences were not verified in the country, and they were not mentioned in the checklist of Iranian mosquitoes [26]. The reports of *Cx. torrentium* in Bushehr [22, 23] and Fars [13] provinces should be verified. This Palearctic species has been confirmed only in northern Iran [26]. Lotfi used unreliable characters to report this species in Fars Province [13]. Differentiating *Cx. torrentium* from *Cx. pipiens* and *Cx. quinquefasciatus* at the larval stage and in adult females is challenging; the most reliable method for verification is examining the male genitalia [26].

### 4.4. Biodiversity and Disease Risk Linkage

Among the present samples, the main vectors of human and animal diseases, such as WNV (*Cx. pipiens, Cx. quinquefasciatus*), dirofilariasis, RVF (*Cx. theileri*), Sindbis, WNV (*Cx. perexiguus*), Japanese encephalitis, RVF, Sindbis, and WNV (*Cx. tritaeniorhynchus*), were available. Both *Cx. pipens* (17%) and *Cx. quinquefasciatus* (12%), which ranked as highly abundant species in our study area, are efficient primary and/or bridge vectors of WNV [39]. There is circumstantial evidence that a few of these species are responsible for the prevailing vector‐borne pathogens in this region.

The genus *Culex* was the first most speciose taxon (11 species, 58%) of all collected genera. Among these, *Cx. pipiens* and *Cx. quinquefasciatus* represented the first and the second most abundant of all the collected *Culex* samples. While the latter southern house mosquito species breeds in polluted water pools rich in organic matter, *Cx. pipiens* thrives in various larval breeding habitats, including septic tanks and sewage wells.

The second most speciose taxon (5 species, 26.3%) of all collected genera belonged to the genus *Anopheles*. Among the five different anopheline species found in Bushehr Province, the proven autochthonous malaria vector species of *An. superpictus* s.l. and *An. stephensi* ranked the first and third in terms of abundance, respectively. In accordance with the findings by others [23], the optimal breeding and replication sites for most *Anopheles* species larvae were sunlit, transparent, nonvegetated water bodies and palm‐irrigated ditches [30, 40]. Although *An. stephensi* is not a “brackish water” specialist, it has been collected from saline‐rich waters off coastal environments [41]. The larvae of this species seldom thrived in brackish cavity habitats, as reported here and in the adjacent Hormozgan Province [40]. Among all collected samples, the saline‐loving *Ae*. *caspius* s.l. was the most abundant species in this province, which is likely related to the brackish water and saline soil of study regions. In the case of *Anopheles* species, the abundance of the high‐salinity tolerant *An. stephensi* was less than that of *An. superpictus* s.l., since the brackish water bodies were patchy and irregular in distribution. Alternatively, it may be postulated that only certain salinity‐tolerant physiologically adaptive *An. stephensi* strains could have the ability to maintain their isotonicity with the external milieu, despite their excretion of water‐insoluble uric acid.

## 5. Public Health Implications

Considering that *Ae. caspius* s.l. was the single most abundant and preponderant (41%) species in all sampling habitats caught during all of the study period in Bushehr Province, it is reasonable to assume its specific and prominent role in the complicated ecological biodiversity of this region. The abundance of this species could be congruous with soil salinity, halophyte cover, and moisture content of its bioecological habitat, since climate in this coastal region is reminiscent of a marine landscape having a relatively high concentration of salt content in its soil. The salinity preference may be an adaptation leading to the decline in competition and predation. This species was the least abundant (0.7%) mosquito present in Khonj County of Fars Province. It is also a highly anthropophagic mosquito that blood‐feeds early morning or late afternoon. In a study from West Azerbaijan Province, northwest Iran, WNV was present in *Ae. caspius* s.l. [42]. It is noteworthy that after more than 7 decades of nonreporting, *Ae*. *aegypti*, an invasive cosmotropical yellow fever mosquito, has recently been found in southern Iran [43, 44]. West Nile virus has also been detected in *Cx. theileri* and *Cx. pipiens* in Iran [45, 46]. This virus has also been isolated from two other rare mosquito species of *Cx. hortensis* and *Cs. longiareolata* [47]. These mosquito species are not mainly anthropophagic, and their viral positivity could be due to ornithophagic activities. It should be noted that specialized methods for collecting invasive *Aedes aegypti* and *Ae. albopictus* such as ovitraps and artificial‐container surveys were not employed in this study, and their absence here does not necessarily imply regional elimination.

As a primary vector for emerging pathogens such as DENV and CHIKV, the resurgence of *Ae. aegypti* in coastal provinces of Iran raises concerns about the ongoing and increasing spread of these highly dangerous arboviruses. Additionally, in southeastern Iran, the potential presence and risk areas of *Ae. albopictus*, the Asian tiger mosquito, a vector of DENV, CHIKV, and Zika virus (ZIKV), have also been investigated [12]. Based on the still uncorroborated finding of six adults and five larvae of *Ae. albopictus* in the Sistan and Baluchistan Province of southeastern Iran in 2016 [37], there is an urgent need to attest this claim. Unfortunately, there are unconfirmed reports of a few new imported fatal human cases due to DENV in southern Iranian provinces during the scorching heat wave of June 2024 (unpublished report). Recent studies have confirmed dengue outbreaks in Sistan‐Baluchestan Province [12, 37], emphasizing the need for continuous monitoring of invasive *Aedes* vectors in southern Iran.

Biodiversity analyses revealed that Tangestan County exhibited the lowest Shannon (1.38) and Simpson (0.0189) indices among all counties in Bushehr Province. These reflected the abundance and evenness of the larval and adult female mosquito species present. So, the low Shannon and Simpson values reflect dominance‐structured mosquito communities typical of saline and environmentally homogeneous habitats, rather than inadequate sampling efforts. Tangestan’s low biodiversity indices likely stem from its homogeneous, saline‐coastal ecosystem, which limits breeding‐site diversity and favors salt‐tolerant species such as *Ae. caspius* s.l., which strongly prevailed the rest of the species present in this county. In contrast, the noncoastal county of Jam presented relatively higher relevant values pertaining to increased diversity and equitability. In this county, *Cx. pipiens* and *Cx. quinquefasciatus* were preponderant over all the other species. On the other hand, the county of Dashti showed a relatively high (0.451) Simpson Index, representing that the probability of diversity is low in this area. The decline in species biodiversity may levitate pathogen transmission and the emergence of infections [6, 7]. Reduced species diversity can increase vector–host contact rates, enhancing disease transmission potential, a pattern consistent with the “dilution effect” hypothesis [7, 8, 10]. The analysis of beta diversity reflected that woodland and littoral habitats were the closest categories in terms of species composition (beta diversity = 2).

The relationship between mosquito biodiversity and disease transmission risk aligns with the “dilution effect” hypothesis, which suggests that reduced biodiversity can amplify pathogen transmission by increasing host–vector contact frequency [7, 8, 10]. Maintaining balanced mosquito and host community structures could therefore contribute to mitigating disease emergence. The observed variation in Shannon and Simpson indices across counties reflects the influence of ecological gradients on mosquito diversity. Higher diversity in Jam and Deylam Counties may result from mixed habitat mosaics and favorable climatic conditions, while lower diversity in Tangestan suggests dominance by salt‐tolerant species adapted to more uniform habitats.

Several vectors of clinically significant pathogens were identified with major health implications. Some of these vectors also transmit infectious agents in neighboring countries, signaling the need for the health system to implement regular surveillance and control programs. This is especially significant during their peak seasons. Careful monitoring of areas where these primary disease vectors are found is essential to prevent their spread and potential outbreaks. Identifying mosquito species diversity allows for a precise assessment of the potential for mosquito‐borne diseases in a specific geographical area, enabling the implementation of integrated vector management (IVM) with a higher likelihood of success.

Habitat preference data indicated that Dashtestan was a “hotspot” county with high mosquito diversity. The complex network of Darwin’s “tangled bank” of species interactions appeared to prevail in this region. Although this county lacked four ecotypes (littoral, arid, saline, and open field), it was densely populated with palm trees and included riverine and cavernicolous habitats. The rivers were rarely flooded except during monsoons, so this ecotype was not included in our data. A major strength of this study was its inclusion of all counties in Bushehr Province and the high catch of mosquito larvae and adults.

The seasonal distribution of adult mosquitoes showed that *Ae. caspius* s.l. peaked in late spring and midsummer, while *Cx. pipiens* abundance extended through autumn. *Anopheles stephensi* populations increased after the first rainfall in late summer, coinciding with the main malaria transmission period in southern Iran. These seasonal patterns are relevant for vector control planning, indicating that control measures should focus on early larval reduction in April–May for *Aedes* and July–September for *Anopheles* species. Strengthening epidemiological surveillance is critical. Integrating entomological monitoring with public health data, particularly for *Culex* and *Aedes* species associated with WNV, DENV, and CHIKV, would provide early warning for outbreaks. Collaboration with the Ministry of Health for routine screening of vector populations is recommended.

### 5.1. Study Limitations

The collection methods employed were optimized for natural and seminatural habitats and adult resting sites; thus, container‐breeding *Aedes aegypti* and *Ae. albopictus* may have been overlooked. No insecticide‐treated bed‐net traps were used. Additionally, Canada balsam mounting can hinder fine morphological discrimination among larval instars. Future studies should include container surveys, molecular identification, and other more suitable larval mounting media. Although geographic latitude and altitude were documented for all counties, a formal latitudinal gradient analysis was not performed because the relatively narrow latitudinal span of Bushehr Province and strong habitat heterogeneity limited its ecological interpretability.

## 6. Conclusions

This study revealed marked spatial heterogeneity in mosquito diversity across Bushehr Province, with *α* diversity ranging from 1.38 to 2.47 and β diversity (Whittaker’s βw) ≈ 1.8. *Ae. caspius* s.l. was the most abundant species, reflecting adaptation to saline habitats. These findings provide baseline data for regional vector‐surveillance programs. We recommend (1) routine monitoring using multiple collection methods; (2) habitat‐specific vector control focusing on high‐abundance zones; and (3) community awareness initiatives to reduce breeding sites. Refining biodiversity analyses with long‐term and molecular data will further enhance vector‐management strategies in southern Iran. Preserving mosquito biodiversity can reduce the incidence of established pathogens. Since a decline in mosquito biodiversity could lead to an increase in disease outcomes, persistent surveillance of vectors and their associated pathogens, effective risk evaluation, and vector control programs would be expected to rely on a renewed comprehensive understanding of mosquito diversity in disparate environments. These findings highlight the need for implementing IVM programs focusing on high‐risk habitats, routine entomological surveillance, and community education campaigns. Preserving ecological balance through sustainable habitat management may reduce vector proliferation and disease risk. By integrating Hill number rarefaction and extrapolation analyses, this study provides a standardized quantitative baseline for future temporal comparisons of mosquito diversity. These modern metrics reduce the influence of collector effort and enhance comparability with global vector‐ecology datasets, ensuring that the study’s findings meet international analytical standards.

## Author Contributions

E.A., H.B., and M.D.M.‐F. conceptualized and wrote the study design and proposal. E.A., H.A., A.D., and M.D. carried out the field collections and identifications. H.A., K.A., and M.D.M.‐F. approved the submitted version. Expert validation on species taxonomy was done by S.A‐H., while S.A‐H., S.A., and M.D.M.‐F. performed data collation and analyses. All authors contributed to the final draft writing and review.

## Funding

This study was supported by Shiraz University of Medical Sciences, 25843.

## Disclosure

An online preprint version of this manuscript is available at “https://papers.ssrn.com/sol3/papers.cfm?abstract_id=5035066”. All authors gave the final approval of this manuscript.

## Ethics Statement

This work was approved after review by the ethics ID: IR.SUMS.SCHEANUT.REC.1401.121 and funded to the corresponding author (M.D.M.‐F.) and supervisor on behalf of his Ph.D. student or the first author (E.A.) by Shiraz University of Medical Sciences (SUMS), Shiraz, Iran.

## Conflicts of Interest

The authors declare no conflicts of interest.

## Data Availability

Data are available on request from the authors.

## References

[1] Eldridge B. F. , Mosquitoes, Encyclopedia of Insects, 2009, Elsevier, 658–663.

[2] Paksa A. , Vahedi M. , Yousefi S. , Saberi N. , Rahimi S. , and Amin M. , Biodiversity of Mosquitoes (Diptera: Culicidae), Vectors of Important Arboviral Diseases at Different Altitudes in the Central Part of Iran, Turkish Journal of Zoology. (2023) 47, no. 2, 111–119, 10.55730/1300-0179.3121.

[3] Harbach R. , Mosquito Taxonomic Inventory, 2024, https://mosquito-taxonomic-inventory.myspecies.info/node/11359.

[4] Azari-Hamidian S. , Norouzi B. , and Harbach R. E. , A Detailed Review of the Mosquitoes (Diptera: Culicidae) of Iran and Their Medical and Veterinary Importance, Acta Tropica. (2019) 194, 106–122, 10.1016/j.actatropica.2019.03.019, 2-s2.0-85063681807. 30898616

[5] Azari-Hamidian S. , Norouzi B. , Maleki H. , Rezvani S. M. , Pourgholami M. , and Oshaghi M. A. , First Record of Aedes (Aedes) cinereus (Diptera: Culicidae) in Iran, Zoology in the Middle East. (2024) 70, no. 2, 136–142, 10.1080/09397140.2024.2359168.

[6] Keesing F. , Belden L. K. , Daszak P. et al., Impacts of Biodiversity on the Emergence and Transmission of Infectious Diseases, Nature. (2010) 468, no. 7324, 647–652, 10.1038/nature09575, 2-s2.0-78649856647. 21124449 PMC7094913

[7] Pongsiri M. J. , Roman J. , Ezenwa V. O. et al., Biodiversity Loss Affects Global Disease Ecology, Bioscience. (2009) 59, no. 11, 945–954, 10.1525/bio.2009.59.11.6, 2-s2.0-77950971416.

[8] Schmidt K. A. and Ostfeld R. S. , Biodiversity and the Dilution Effect in Disease Ecology, Ecology. (2001) 82, no. 3, 609–619, 10.1890/0012-9658(2001)082[0609:batdei]2.0.co;2, 2-s2.0-0035104467.

[9] Vasmehjani A. A. , Rezaei F. , Farahmand M. et al., Epidemiological Evidence of Mosquito-Borne Viruses Among Persons and Vectors in Iran: A Study from North to South, Virologica Sinica. (2022) 37, no. 1. 10.1016/j.virs.2022.01.005 PMC8922425 35234614

[10] Foster W. A. and Walker E. D. , Mosquitoes (Culicidae), Medical and Veterinary Entomology, 2019, Elsevier, 261–325.

[11] Azari-hamidian S. and Harbach R. , Arthropod-Borne and Arthropod-Related Viruses in Iran and Neighboring Countries, Parazitologiâ. (2023) 57, no. 5, 356–440, 10.31857/s0031184723050010.

[12] Nejati J. , Bueno-Mari R. , Collantes F. et al., Potential Risk Areas of *Aedes albopictus* in South-Eastern Iran: A Vector of Dengue Fever, Zika, and Chikungunya, Frontiers in Microbiology. (2017) 8, 10.3389/fmicb.2017.01660, 2-s2.0-85028755151. PMC5591785 28928720

[13] Lotfi M. D. , Iranian Species of Genus Culex (Culinae:Diptera), Bull Soc Pathol Exot Filiales. (1970) 63, no. 3, 399–403. 5537819

[14] Macan T. T. , The Anopheline Mosquitoes of Iraq and North Persia, 1950, H. K. Lewis & Co., Ltd, London.

[15] Dow R. P. , Notes on Iranian Mosquitoes, American Journal of Tropical Medicine and Hygiene. (1953) 2, no. 4, 683–695, 10.4269/ajtmh.1953.2.683, 2-s2.0-0348009147. 13065637

[16] Eshghi N. , Motabar M. , Javadian E. , and Manoutcheri A. , Biological Features of *Anopheles fluviatilis* and Its Role in the Transmission of Malaria in Iran, Tropical & Geographical Medicine. (1976) 28, no. 1, 41–44. 941241

[17] Edrissian G. H. , Manouchehry A. V. , and Hafizi A. , Application of an Enzyme-Linked Immunosorbent Assay (ELISA) for Determination of the Human Blood Index in Anopheline Mosquitoes Collected in Iran, Journal of the American Mosquito Control Association. (1985) 1, no. 3, 349–352. 3880251

[18] Saebi M. , Morphological Study on Anopheline Larvae and Their Distribution in Iran, 1987, School of Public Health, Tehran University of Medical Sciences, Tehran, Tehran, Iran.

[19] Zaim M. , The Distribution and Larval Habitat Characteristics of Iranian Culicinae, Journal of the American Mosquito Control Association. (1987) 3, no. 4, 568–573. 2904967

[20] Harbach R. E. , The Mosquitoes of the Subgenus Culex in Southwestern Asia and Egypt (Diptera: Culicidae), Contributions of the American Entomological Institute. (1988) 24, no. 1.

[21] Dehghan H. , Sadraei J. , Moosa-Kazemi S. H. , Baniani N. A. , and Nowruzi F. , The Molecular and Morphological Variations of *Culex pipiens* Complex (Diptera: Culicidae) in Iran, Journal of Vector Borne Diseases. (2013) 50, no. 2, 111–120, 10.4103/0972-9062.117482. 23995312

[22] Khoobdel M. , Keshavarzi D. , Mossa-Kazemi S. H. , and Sobati H. , Species Diversity of Mosquitoes of the Genus Culex (Diptera, Culicidae) in the Coastal Areas of the Persian Gulf, AIMS Public Health. (2019) 6, no. 2, 99–106, 10.3934/publichealth.2019.2.99. 31297396 PMC6606530

[23] Khoobdel M. , Keshavarzi D. , Sobati H. , and Akbari M. , Species Diversity, Habitat and Abundance of Culicid Mosquitoes in Bushehr Province, South of Iran, Biodiversitas. (2020) 21, no. 4, 1401–1406.

[24] Dehghani A. , Lotfi M. H. , Falahzadeh H. , Vahdat K. , and Shabani Z. , Epidemiological Study and Spatial Modeling of Cutaneous Leishmaniasis in Bushehr Province Using the Geographic Information System (GIS) from 2011 to 2015, J Commun Health Res. (2019) 8, no. 3, 156–163.

[25] Cheghabalaki Z. Z. , Yarahmadi D. , Karampour M. , and Shamsipour A. , Spatial Dynamics of a Phlebotomine Sand Flies Population in Response to Climatic Conditions in Bushehr Province of Iran, Annals of Global Health. (2019) 85, no. 1, 10.5334/aogh.30, 2-s2.0-85065440787. PMC6634292 31025839

[26] Azari-Hamidian S. and Harbach R. E. , Keys to the Adult Females and fourth-instar Larvae of the Mosquitoes of Iran (Diptera: Culicidae), Zootaxa. (2009) 2078, no. 1, 1–33, 10.11646/zootaxa.2078.1.1.

[27] Simpson E. , Measurement of Diversity, Nature. (1949) 163, no. 4148, 10.1038/163688a0, 2-s2.0-33344464667.

[28] Hsieh T. , Ma K. , and Chao A. , iNEXT: An R Package for Rarefaction and Extrapolation of Species Diversity (Hill Numbers), Methods in Ecology and Evolution. (2016) 7, no. 12, 1451–1456, 10.1111/2041-210x.12613, 2-s2.0-84992119898.

[29] Chao A. , Gotelli N. J. , Hsieh T. et al., Rarefaction and Extrapolation with Hill Numbers: a Framework for Sampling and Estimation in Species Diversity Studies, J Ecological monographs. (2014) 84, no. 1, 45–67, 10.1890/13-0133.1, 2-s2.0-84893283120.

[30] Omrani S.-M. and Azarihamidian S. , Vertical Distribution, Biodiversity, and Some Selective Aspects of the Physicochemical Characteristics of the Larval Habitats of Mosquitoes (Diptera: Culicidae) in Chaharmahal and Bakhtiari Province, Iran, International Journal of Epidemiologic Research. (2020) 7, no. 2, 74–91, 10.34172/ijer.2020.15.

[31] Soltani Z. , Keshavarzi D. , Ebrahimi M. et al., The Fauna and Active Season of Mosquitoes in West of Fars Province, Southwest of Iran, Arch Razi Inst. (2017) 72, no. 3, 203–208, 10.22092/ari.2017.111603, 2-s2.0-85029764808. 30341942

[32] Keshavarzi D. , Soltani Z. , Ebrahimi M. et al., Monthly Prevalence and Diversity of Mosquitoes (Diptera: Culicidae) in Fars Province, Southern Iran, Asian Pac J Trop Dis. (2017) 7, no. 2, 112–120, 10.12980/apjtd.7.2017d6-369, 2-s2.0-85020241932.

[33] Moosa-Kazemi S. , Vatandoost H. , Nikookar H. , and Fathian M. , Culicinae (Diptera: Culicidae) Mosquitoes in Chabahar County, Sistan and Baluchistan Province, Southeastern Iran, Iranian Journal of Arthropod-Borne Diseases. (2009) 3, no. 1, 29–35. 22808369 PMC3385524

[34] Jaberhashemi S. A. , Azari-Hamidian S. , Soltani A. et al., The Fauna, Diversity, and Bionomics of Culicinae (Diptera: Culicidae) in Hormozgan Province, Southern Iran, Journal of Medical Entomology. (2022) 59, no. 3, 987–996, 10.1093/jme/tjac003. 35134206

[35] Poudat A. , Edalat H. , Zaim M. , Rezaei F. , Abadi Y. S. , and Basseri H. R. , Species Composition and Geographic Distribution of Culicinae Mosquitoes and Their Possible Infection with West Nile Virus in Hormozgan Province, Southern Iran, Iranian Journal of Public Health. (2023) 52, no. 4, 809–817, 10.18502/ijph.v52i4.12454. 37551191 PMC10404324

[36] Nejati J. , Azari-Hamidian S. , Oshaghi M. A. , Vatandoost H. , White V. L. , Moosa-Kazemi S. H. , Bueno-Marí R. , Hanafi-Bojd A. A. , Endersby-Harshman N. M. , Axford J. K. , Karimian F. , Koosha M. , Choubdar N. , and Hoffmann A. A. , The monsoon-associated Equine South African Pointy Mosquito ‘Aedes Caballus’; the First Comprehensive Record from Southeastern Iran with a Description of Ecological, Morphological, and Molecular Aspects, PLoS One. (2024) 19, no. 5, 10.1371/journal.pone.0298412. PMC11115297 38781219

[37] Doosti S. , Yaghoobi-Ershadi M. R. , Schaffner F. et al., Mosquito Surveillance and the First Record of the Invasive Mosquito Species Aedes (Stegomyia) Albopictus (Skuse) (Diptera: Culicidae) in Southern Iran, Iranian Journal of Public Health. (2016) 45, no. 8, 1064–1073. 27928533 PMC5139964

[38] Dorzaban H. , Soltani A. , Alipour H. et al., Mosquito Surveillance and the First Record of Morphological and Molecular-based Identification of Invasive Species Aedes (Stegomyia) Aegypti (Diptera: Culicidae), Southern Iran, Experimental Parasitology. (2022) 236-237, 10.1016/j.exppara.2022.108235. 35247382

[39] Hamer G. L. , Kitron U. D. , Brawn J. D. et al., *Culex pipiens* (Diptera: Culicidae): a Bridge Vector of West Nile Virus to Humans, Journal of Medical Entomology. (2008) 45, no. 1, 125–128, 10.1093/jmedent/45.1.125. 18283952

[40] Vatandoost H. , Oshaghi M. A. , Abaie M. R. et al., Bionomics of *Anopheles stephensi* Liston in the Malarious Area of Hormozgan Province, Southern Iran, 2002, Acta Tropica. (2006) 97, no. 2, 196–203, 10.1016/j.actatropica.2005.11.002, 2-s2.0-30644465967. 16329986

[41] Taylor R. , Messenger L. A. , Abeku T. A. , Clarke S. E. , Yadav R. S. , and Lines J. , Invasive *Anopheles stephensi* in Africa: Insights from Asia, Trends in Parasitology. (2024) 40, no. 8, 731–743, 10.1016/j.pt.2024.06.008. 39054167

[42] Bagheri M. , Terenius O. , Oshaghi M. A. et al., West Nile Virus in Mosquitoes of Iranian Wetlands, Vector Borne and Zoonotic Diseases. (2015) 15, no. 12, 750–754, 10.1089/vbz.2015.1778, 2-s2.0-84951768381. 26565610

[43] Azari-Hamidian S. , The Invasive Aedes Mosquitoes (Diptera: Culicidae) and Their Medical and Veterinary Importance: A Mini Review, Caspian Journal of Health Research. (2023) 8, no. 4, 241–246.

[44] Paksa A. , Azizi K. , Yousefi S. , Dabaghmanesh S. , Shahabi S. , and Sanei-Dehkordi A. , First Report on the Molecular Phylogenetics and Population Genetics of *Aedes aegypti* in Iran, Parasites & Vectors. (2024) 17, no. 1, 10.1186/s13071-024-06138-3. PMC10835860 38303048

[45] Shahhosseini N. , Chinikar S. , Moosa‐Kazemi S. H. et al., West Nile Virus Lineage‐2 in Culex Specimens from Iran, Tropical Medicine and International Health. (2017) 22, no. 10, 1343–1349, 10.1111/tmi.12935, 2-s2.0-85030625831. 28746985

[46] Shahhosseini N. , Moosa-Kazemi S. H. , Sedaghat M. M. et al., Autochthonous Transmission of West Nile Virus by a New Vector in Iran, vector-host Interaction Modeling and Virulence Gene Determinants, Viruses. (2020) 12, no. 12, 10.3390/v12121449. PMC7766443 33339336

[47] Khaledian M. , Owliaee I. , Sazmand A. , Davari B. , Zahirnia A. H. , and Jalilian F. A. , West Nile Virus in Adults and Larvae of *Culiseta longiareolata* and *Culex hortensis* (Diptera: Culicidae) Captured in Hamedan, Western Iran, Acta Tropica. (2024) 260, 10.1016/j.actatropica.2024.107434. 39413896

